# Withdrawal of mechanical ventilation in amyotrophic lateral sclerosis patients: a multicenter Italian survey

**DOI:** 10.1007/s10072-023-06905-7

**Published:** 2023-07-07

**Authors:** Cristina Moglia, Francesca Palumbo, Simone Veronese, M. N. D. Italian Study Group, Stefania Angelocola, Paolo Barone, Ilaria Bartolomei, Enrica Bersano, Alessandro Bombaci, Giuseppe Borghero, Sara Cabras, Chiara Cambieri, Elena Canali, Antonio Canosa, Margherita Capasso, Claudia Caponnetto, Patrizio Cardinali, Mario Casmiro, Marco Ceccanti, Adriano Chiò, Monica Consonni, Eleonora Dalla Bella, Fabiola De Marchi, Filippo De Mattei, Eustachio D’Errico, Francesca Di Pede, Luca Diamanti, Raffaele Dubbioso, Massimo Filippi, Massimiliano Filosto, Nicola Fini, Giulia Gianferrari, Maurizio Grassano, Maurizio Inghilleri, Vincenzo La Bella, Giuseppe Lauria Pinter, Laura Libonati, Francesco Logullo, Jessica Mandrioli, Umberto Manera, Ilaria Martinelli, Gioacchino Martusciello, Sabrina Matà, Enrico Matteoni, Letizia Mazzini, Doriana Medici, Stefania Miniello, Federica Moret, Cecilia Nozzoli, Giovanni Piccirillo, Giovanna Pilurzi, Nilo Riva, Silvia Romito, Massimo Russo, Fabrizio Salvi, Elisabetta Sette, Vincenzo Silani, Isabella Laura Simone, Cecilia Simonini, Rossella Spataro, Giovanna Squintani, Salvatore Stano, Raffaella Tanel, Gioacchino Tedeschi, Nicola Ticozzi, Antonella Toriello, Lucio Tremolizzo, Francesca Trojsi, Veria Vacchiano, Rosario Vasta, Paolo Volanti, Lucia Zinno, Elisabetta Zucchi, Andrea Calvo

**Affiliations:** 1 “Rita Levi Montalcini”, Department of Neuroscience, ALS Centre, University of Turin, Turin, Italy; 2 Azienda Ospedaliero-Universitaria Città Della Salute e Della Scienza Di Torino, Turin, Italy; 3 Fondazione FARO, Turin, Italy

**Keywords:** Amyotrophic lateral sclerosis, Law 219/2017, Mechanical ventilation withdrawal, End-of-life care, Palliative care, Advance care planning

## Abstract

**Background:**

Law 219/2017 was approved in Italy in December 2017, after a years-long debate on the autonomy of healthcare choices. This Law, for the first time in Italian legislation, guarantees the patient’s right to request for withdrawal of life-sustaining treatments, including mechanical ventilation (MV).

**Objective:**

To investigate the current status of MV withdrawal in amyotrophic lateral sclerosis (ALS) patients in Italy and to assess the impact of Law 219/2017 on this practice.

**Methods:**

We conducted a Web-based survey, addressed to Italian neurologists with expertise in ALS care, and members of the Motor Neuron Disease Study Group of the Italian Society of Neurology.

**Results:**

Out of 40 ALS Italian centers, 34 (85.0%) responded to the survey. Law 219/2017 was followed by an increasing trend in MV withdrawals, and a significant increase of neurologists involved in this procedure (*p* 0.004). However, variations across Italian ALS centers were observed, regarding the inconsistent involvement of community health services and palliative care (PC) services, and the intervention and composition of the multidisciplinary team.

**Conclusions:**

Law 219/2017 has had a positive impact on the practice of MV withdrawal in ALS patients in Italy. The recent growing public attention on end-of-life care choices, along with the cultural and social changes in Italy, requires further regulatory frameworks that strengthen tools for self-determination, increased investment of resources in community and PC health services, and practical recommendations and guidelines for health workers involved.

**Supplementary Information:**

The online version contains supplementary material available at 10.1007/s10072-023-06905-7.

## Introduction

Respiratory failure is responsible for the majority of deaths from amyotrophic lateral sclerosis (ALS) [1], and it can be treated with noninvasive mechanical ventilation (NIMV) and/or invasive mechanical ventilation (IMV) via tracheostomy [2]. NIMV and IMV have been shown to improve respiratory symptoms and increase survival [3, 4]. NIMV has also been shown to improve quality of life of ALS patients [5]. However, as the disease progresses, dependence on respiratory support increases, which in some cases may lead the patients to require discontinuation of assisted ventilation because the burdens of ventilation outweigh the benefits. In Italy, in January 2018, Law 219/2017 “Provisions for informed consent and advance directives treatment” [6] entered into force, at the end of a fierce cultural, social, and political debate on end-of-life care, particularly on the right to refuse potentially life-saving treatments. Law 219/2017 states that no medical treatment can be initiated or continued without the patient’s free and informed consent, even in the case of life-sustaining treatments, such as mechanical ventilation (MV) [7]. To date, it is not known how frequent the request for suspension of MV is in ALS patients in Italy, and even less is known about the procedural steps applied and whether Law 219/2017 has had any impact on this practice. The present study aimed to investigate the current status of the MV withdrawal in ALS patients in Italy and to assess the impact of Law 219/2017 on this procedure.

## Materials and methods

### Data collection

We conducted a multicenter Web-based anonymous survey between January and May 2020. An email invitation to fill out an electronic form was addressed to Italian neurologists with expertise in ALS care, and members of the Motor Neuron Disease Study Group of the Italian Society of Neurology.

The survey consisted of 11 questions (Table 1). To minimize response bias, the survey was administered in Italian, was anonymous, open for more than 4 months and was composed of various types of questions (yes/no questions, multiple choice questions, and open questions). Furthermore, each response, even in the case of responses from the same ALS center, was independently analyzed. In cases where a center provided one response, it was considered as representative of that center, assuming that the management of ALS patients within the same center is uniform. To evaluate the possible impact of the geographic area on the answers, we divided the centers into two groups (north Italy ALS centers and central-south Italy ALS centers) and performed a comparative analysis. Consent was assumed when the participant started the survey. Data provided by respondents were collected in the online platform and exported for statistical analysis.

**Table 1:** Questions and answers of the survey

	Total answers (*n* = 38)	
	Yes	No
1. Have you ever received a request for MV withdrawal before or after Law 219/2017?	22 (57.9%)	16 (42.1%)
1-a. Was the MV withdrawal completed?	16 (72.7%)	6 (27.3%)
1-b. Was the request managed by a MDT?	16 (72.7%)	6 (27.3%)
1-c. Which was the setting of the MV withdrawal?	Home, 9 (41.3%); hospital, 7 (30.4%); hospice, 6 (28.2%)	
1-d. What was the duration of the whole procedure?	Mean: 3.6 ± 2.4 months; interval: 1 week–6 months	
1-e. Which were the decisional steps applied to manage the request?	Discussion with patients and family to assess the autonomy and awareness of the request, reassessment of the request after a variable time, collection of the patient’s wishes, MV withdrawal planning, 22 (100%); seeking medical-legal and ethical support before initiating the procedure, 2 (9.0%)	
	Yes	No
2. Have you ever taken part in a MV withdrawal (as organizer, performer, or observer) before or after Law 219/2017?	25 (65.7%)	13 (34.3%)
2-a. In this case, was ACP previously discussed?	23 (92.0%)	2 (8.0%)
3. Have you ever refused or deemed inappropriate a MV withdrawal request?	0 (0.0%)	22 (100%)
22. In the case of a MV withdrawal request, is there a MDT at your center?	27 (71.0%)	11 (29%)
4. a- In this case, who are the members of the MDT, apart from the neurologist?	Psychologist, 19 (70.3%); PM physician, 12 (44.4%); pulmonologist, 9 (33.3%); anesthesiologist, 8 (29.6%); general practitioner, 6 (22.2%); nurse, 7 (25.9%); psychiatrist, 3 (11.1%); medical and legal adviser of the hospital and bioethicist, 2 (7.4%)	

Table showing questions and answers of the survey on MV withdrawals in ALS patients in Italy

*ALS*, amyotrophic lateral sclerosis; *MV*, mechanical ventilation; *MDT*, multidisciplinary team; *ACP*, advance care planning; *PM*, palliative medicine

### Statistical analysis

Descriptive statistics (mean, range for dimensional data, and proportions for dichotomous data) were used to summarize the results. Categorical variables were reported as percentages. Chi-squared test and Fisher’s exact test were used to compare proportions. The 2-tailed significance level was set at *p* < 0.05. Data were analyzed using the Statistical Package for Social Sciences (SPSS version 25.0 for Windows, Chicago, IL, 2017).

### Ethical approval

This study was approved by the local ethics committee Comitato Bioetica d’Ateneo Università degli Studi di Torino (protocol no. 0486598, 30/07/2021). The study was performed in accordance with World Medical Association Declaration of Helsinki.

## Results

Thirty-eight responses were collected. Thirty-four out of 40 Italian ALS centers provided at least one response, so a response rate of 85.0% was obtained. Thirty centers (88.2%) provided one response and 4 (11.7%) centers provided two responses.

In 22 (57.9%) centers, at least one request for MV withdrawal had been received. This request was followed in all cases by a discussion with patient and caregivers, and by the assessment of patient’s cognitive and emotional state. This evaluation was performed in 16 (72.7%) cases by a psychologist, in 3 (13.6%) cases by a psychologist and a psychiatrist, in 2 (10.5%) cases by the neurologist, and in 1 (4.5%) case by the palliative medicine (PM) physician.

In 16 cases (72.7%), the procedure was managed by a multidisciplinary team (MDT). In general, the presence of a MDT in case of MV withdrawal request was reported in 27 (71.0%) cases. In addition to neurologist, the other health workers involved in the composition of the MDT were as follows: PM physician in 12 (44.4%) cases, pulmonologist in 9 (33.3%) cases, anesthesiologist in 8 (29.6%) cases, general practitioner in 6 (22.2%) cases, nurse in 7 (25.9%) cases, psychologist in 19 (70.3%) cases, psychiatrist in 3 (11.1%) cases, and the medical and legal advisers of the hospital and bioethicist in 2 (7.4%) cases. In the latter two cases, both occurred prior to the Law 219/2017, medical and legal adviser were involved, as ethical and legal support were sought before starting the procedure. The main differences in the composition of the MDT between north Italy and central-south Italy ALS centers regarded the PM Specialist, involved in 8 (50.0%) vs 4 (36.3%) cases (*p* 0.69); the nurse, involved in 5 (31.2%) vs 2 (18.1%) cases (*p* 0.66); the psychologist, involved in 12 (75.0%) vs 7 (63.6%) cases (*p* 0.67), and the psychiatrist, involved in 3 (18.75%) vs no cases (*p* 0.24), respectively (Supplementary Material-1).

The decision was then reevaluated after a variable period of time. If the decision was appropriate, conscious, and remained unchanged, the MV withdrawal was planned.

**Fig. 1:**
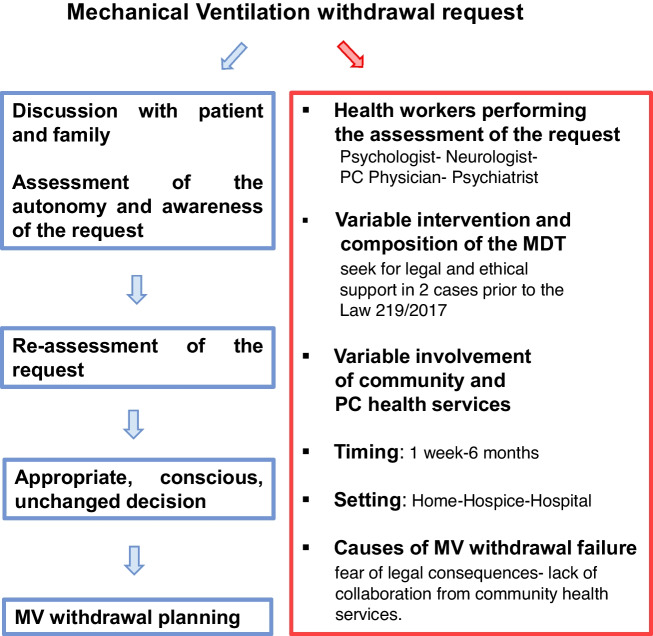
Picture showing the similarities (in blue, left) and variabilities (in red, right) in the management of a MV withdrawal request across Italian ALS centers. Abbreviations: MV, mechanical ventilation; MDT, multidisciplinary team; PC, palliative care

In 22 cases (100%), the MV withdrawal request was deemed appropriate and, among these, MV withdrawal was practiced in 16 (72.7%) cases. In one case, the patient recanted his wish to withdraw MV; in a second case, the patient’s death occurred during the process of evaluation of the request. The duration of the whole procedure ranged from one week to a maximum of 6 months, with a mean time of 3.6 months. The setting was home in 9 (41.3%) cases, hospital in 7 (30.4%) cases, and hospice in 6 (28.2%) cases (Fig. 1).

### Impact of Law 219/2017 on MV withdrawal

**Fig. 2:**
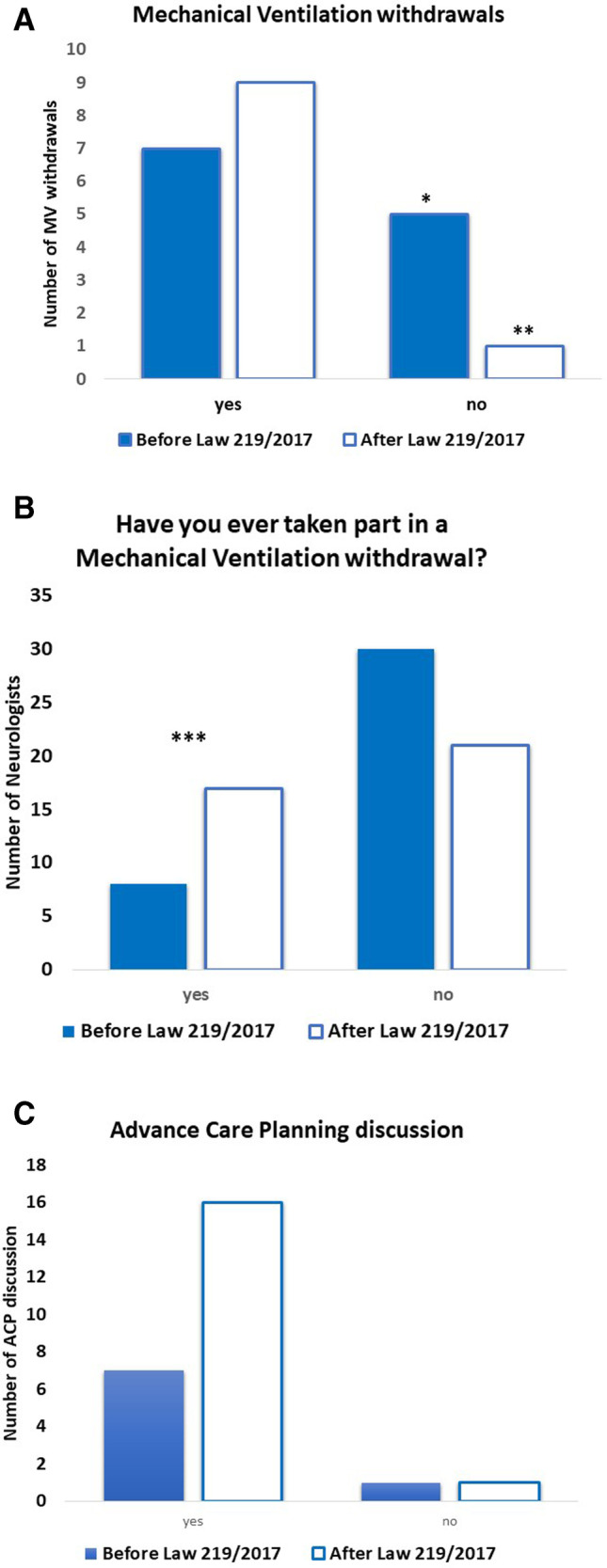
**A** Mechanical ventilation withdrawals performed before and after Law 219/2017. *In one case patient’s death occurred during the evaluation of the request. **In one case the patient recanted his wish to withdraw MV, in a second case the request for MV withdrawal was under evaluation at the time of the survey. **B** Neurologists’ involvement in a mechanical ventilation withdrawal before and after Law 219/2017. ***Statistically significant increase. **C** Advance care planning discussion before and after Law 219/2017. Abbreviations: MV, mechanical ventilation; ACP, advance care planning

Before Law 219/2017, MV withdrawals were practiced in 7 out of 12 cases (58.3%). Two requests were not complied with due to “the lack of cooperation from local medical services” and “the fear of legal consequences.” In 1 (8.3%) case, the patient’s death occurred during the evaluation of the request, and in 1 (8.3%) case, the patient recanted his wish to withdraw MV. After the Law, MV withdrawals were practiced in 9 out of 10 (90.0%) cases, and in 1 case (10%), the request was still being evaluated (*p* 0.16) (Fig. 2A). The percentage of neurologists involved in organizing, participating in, or performing a MV withdrawal, increased from 8 (21.0%) to 17 (44.7%) after Law (*p* 0.004) (Fig. 2B). In cases where the neurologists took part in the procedure, advance care planning (ACP) was discussed in 23 (92.0%) cases. Specifically, in 7 out of 8 (87.5%) cases before Law, and in 16 out of 17 (94.1%) cases after Law (*p* 0.32) (Fig. 2C, Table 2, Supplementary Material-2).

**Table 2:** MV withdrawals, neurologists’ involvement, and ACP discussion before vs after Law 219/2017

	Before Law 219/2017	After Law 219/2017	*p*
MV withdrawals requests	12	10	
MV withdrawals completed	7 (58.3%)	9 (90.0%)	0.16
Neurologists’ involvement in a MV withdrawal (as organizer, performer or observer)	8/38 (21.0%)	17/38 (44.7%)	**0.004**
ACP discussion (in case of neurologist’s involvement)	7 (87.5%)	16 (94.1%)	0.32

Table showing the comparison of MV withdrawals, neurologists’ involvement, and ACP discussion before and after Law 219/2017. Significant *p* values are reported in bold

*MV*, mechanical ventilation; *ACP*, advance care planning

## Discussion

The present survey aimed to assess the current status of MV withdrawal in ALS patients in Italy and to evaluate the impact of Law 219/2017 on this practice. According to our results, 57.9% of neurologists responding to the survey received at least one request for MV withdrawal in their clinical practice and it was performed in 72.7% of cases.

As expected, we observed overall similarities with regard to the key procedural steps outlined in the current guidelines on MV withdrawal in motor-neuron disease [8–11]. Specifically, the assessment of the request with patient and families to ascertain the autonomy of the decision and the awareness of its consequences; the reassessment of the decision to verify if it remained unchanged; the MV withdrawal planning if the decision was deemed appropriate and conscious. The intervention of a MDT was reported in 72.7% of the cases, and, most notably, it was the same percentage of MV withdrawal complied with, as mentioned above. The high percentage of MDT involvement and the overlap with MV withdrawals are both positive results, although needing confirmation on larger samples. It is known that a MDT approach is useful to address ALS patients’ broad range of needs throughout the course of the disease [12], and it would seem reasonable to deem it effective on the management of end-of-life issues, such as a MV withdrawal.

Variability across centers was observed with regard to the composition of the MDT, which, in addition to the neurologist, was composed of general practitioners only in 22% of cases and PM specialists in 44% of cases. Other health workers variably involved were pulmonologists, anesthesiologists, nurses, psychologists, and psychiatrists. Furthermore, the health workers involved in the first assessment of the request varied across centers. In 72.7% of cases, it was carried out by the psychologist, in 13.6% of cases by the psychologist together with psychiatrist, in 15% of cases by the neurologist or PM physician. Then, in essence, there was the lack of a standard decision-making and emotional assessment as part of evaluation of the request. From a practical point of view, it is quite expected that this assessment may be variably performed by the physician receiving the request, and only in some cases by the psychologist and/or the psychiatrist, in order to detect an eventual and treatable mental-health disorder or acute mood disturbance. However, taking into account that a cognitive and/or emotional impairment can arise during the course of the disease in up to 50% of ALS patients and that this is not necessarily detected without a targeted assessment [13, 14], the involvement of neuropsychological and psychiatric assessment after a MV withdrawal request is made should be considered.

The inconsistent involvement of general practitioners is in keeping with previous results showing the limited integration between end-of-life care and community health services in Italy [15] and it is also coherent with the result of a prevalent (58.6%) hospital or hospice setting for the MV withdrawal versus home-setting (41.3%), showing it was not possible to conduct the procedure in a home-setting in more than half of the cases.

Moreover, in one case of non-completion of MV withdrawal, the reported cause was the difficulty of coordination with local medical services. Noteworthily, the collaboration of neurologists with PM specialists occurred in less than half of the cases, and the central-south Italy ALS centers showed less cooperation with PM specialist compared to north Italy ones, although not reaching statistical significance. This also applied to the psychiatrist, psychologist and nurse. The variety of the health workers involved in the composition of the MDT may be due to several reasons. Firstly, the different working conditions and resources investment in health services across Italian regions, often showing a north–south gradient [16, 17]. At the national level, there are currently significant differences in the density of palliative care (PC) network and many of the Italian territories are not adequately served [18], although PC Services offer a great deal for these patients’ symptoms management [19–21], are considered by the Italian legislation as one of the “Essential Levels of Care” (Law 38/2010) [22], and are recommended in several guidelines for ALS from the early stages of the disease [9, 12].

Cultural factors may also play a role. In fact, there is often a reluctance of patients and families for referral to the PM specialist, associating this with imminent death and lack of hope [23, 24], and this may represent a very important barrier to an effective collaboration. A similar attitude is also observed toward the psychiatrist and the psychologist, typically associated with mental illness, still carrying a stigma in Italian culture [25–27]. Moreover, families and caregivers often struggle to recognize the cognitive or emotional deterioration caused by the disease, and are reluctant to accept the usefulness of a psychological support for themselves. To date, several guidelines highlight the crucial role of the psychologist in the end-of-life care [28–30], as they facilitate the conversation addressing the stigma around death and dying, help the process of decision-making, and provide a support to patients and families to cope with stress, loss, and changes in identity, all of which apply in end-of-life [31]. In this role, the psychologist performs the function of the specialist palliative care social worker, that is absent in Italy, unlike other Western countries [32].

Another cause of heterogeneity may be the presence of practical obstacles to a multidisciplinary management, also related to differences in terms of effectiveness of community health services across Italian regions [16]. Specifically, differentiated performances have been observed not only between north and south, but also between northeast and northwest, as well as among regions of the center [33]. Noteworthily, a recent task force of the Italian College of General Practitioners and Primary Care has been formed, with the aim of a greater integration with palliative care services, including end-of-life [34]*.* This is an important initiative with a potential impact on end-of-life care of ALS patients, particularly as home is the preferred place of death in the majority of terminal ill patients [35] and about 60% of the Italian citizens [36].

One last cause of heterogeneity is certainly the lack of standard protocols and practical recommendations on the issue, also due to the fairly recent entry into force of the Law 219/2017.

In this perspective, clinical guidelines encouraging a collaborative approach may be useful. Furthermore, a continuing PC training and education for all physicians, included neurologists, is needed, as Italian neurologists often lack of an adequate education and training in PC [12], as recommended by the EAN/EAPC consensus on palliative care [37], and required by the Law 219/2017 [6]. Education is also needed for patients and families, so that there is a clear understanding that a psychological support may be beneficial for all involved, and that palliative care may be useful at any stage of ALS [32].

Finally, we would like to point out the lack of references to the spiritual consultant. This is likely related to the fact that spiritual care in Italy is conceived as intrinsically associated with religion and the need for spiritual support considered a personal event, untied from healthcare, and for which direct contact is made with a religious official. However, recently there has been a growing attention on this issue, and several initiatives have been conducted to integrate spiritual care with palliative care [38–40].

As regard the impact of the Law 219/2017, our results show that the entry into force of the Law 219/2017 had an overall positive impact on MV withdrawal in ALS, although certainly needing confirmation on larger sample. In fact, the entry into force of the Law was followed by a significant increase of neurologists involved in the procedure as organizer, executor, or observer. Moreover, the frequency of MV suspensions and ACP discussions showed an upward trend after the entry into force of the Law. For two cases before the Law was passed ethical and legal support from medical and legal advisers of the hospital and a bioethicist was sought, but this was never reported after the Law. Moreover, one of the causes of MV withdrawal failure before Law was the fear of legal consequences, while after Law this concern was never reported. This likely reflects the fact that the Law represents a help in daily medical practice and significantly decreases the risk of professional medical liability [41, 42]. Furthermore, in no case the request for MV withdrawal was rejected or deemed inappropriate a priori by neurologists, and in all cases, it has been evaluated, both before and after Law, showing an overall positive attitude of neurologists toward a request for self-determination in end-of-life choices, as previously reported [12].

One of the limitations of this work is that not all Italian ALS centers responded to the survey, limiting the generalizability of the obtained results. The recent entry into force of the Law 219/2017 limits the interpretation of the results obtained comparing the period before and after Law. The small sample size led to the consideration of the neurologists as a single group and so it was not possible to differentiate between organizing, performing, and observing a MV withdrawal procedure. Further studies are needed on this little explored and yet crucial field of care, also involving patients, and other health workers, such as PM specialists and general practitioners, to outline the practical obstacles that are encountered in end-of-life care clinical practice and to highlight the strengths and limits of the current legislation on the subject.

Italy represents a controversial scenario on end-of-life care, since the debate on healthcare choices has developed later compared to many other Western countries [43]. However, in the last years, there has been a cultural shifting that has led to the increase of public attention on end-of-life issues, and after the sentence 242/2019 of the Constitutional Court, it is possible to request medically assisted suicide for patients affected by an irreversible pathology, surviving due to life-saving treatments, and who are shown to be capable of understanding and willing [44].

In conclusion, Law 219/2017 represented a step-forward toward the right to self-determination in Italy and our results show that the entry into force of the Law was followed by an increasing trend in the frequency of MV withdrawals in ALS patients and a significant increase of neurologists involved in this procedure. Some variations were observed among Italian ALS centers with regard to the health workers involved, the collaboration with health community, and the involvement of PC services. The recent and growing public attention around issues of self-determination and the cultural change in social and professional relations require regulation of this area of care, including for those patients for whom discontinuation of life-support therapies is not sufficient to end their suffering. In addition to regulatory frameworks, the development of strong end-of-life practices requires education and training, increased resources for community and PC health services, and practical recommendations and guidelines for the health workers involved.

### Supplementary Information

Below is the link to the electronic supplementary material.

Supplementary file1 (DOCX 23 KB)

Supplementary file2 (DOCX 31 KB)

## Data Availability

Data will be available upon request by interested researchers.
