# Supplementary material for: Withdrawal of mechanical ventilation in amyotrophic lateral sclerosis patients: a multicenter Italian survey
Source: Neurol Sci. 2023 Jul 7;44(12):4349–57. doi: 10.1007/s10072-023-06905-7 (PMC10641048; doi:10.1007/s10072-023-06905-7)
Supplement: Supplementary file 1 — Supplementary file1 (DOCX 23 KB) [file 10072_2023_6905_MOESM1_ESM.docx]

| Supplementary Material 1-  Health workers involved in the MDT: North Italy vs Central-South Italy ALS Centers | | | |
| --- | --- | --- | --- |
|  | **North Italy**  **ALS Centers**  **(total answers =22)** | **Central-South Italy**  **ALS Centers**  **(total answers=16)** | **p** |
| MDT (YES/NO) | 16 (72.72%)/  6 (27-27%) | 11 (68.75%)/  5 (31.25%) | 0.74 |
| Health workers of the MDT |  |  |  |
| PM Specialist | 8 (50.00%) | 4 (36.36%) | 0.69 |
| Pulmonologist | 6 (37.50%) | 3 (27.27%) | 0.69 |
| Anesthesiologist | 3 (18.75%) | 5 (45.45%) | 0.20 |
| General Practitioner | 4 (25.00%) | 2 (18.18%) | 0.66 |
| Nurse | 5 (31.25%) | 2 (18.18%) | 0.68 |
| Psychologist | 12 (75.00%) | 7 (63.63%) | 0.67 |
| Psychiatrist | 3 (18.75%) | 0 (0.00%) | 0.24 |
| Medical and Legal adviser of the Hospital | 2 (12.50%) | 0 (0.00%) | 0.49 |
| Bioethicist | 2 (12.50%) | 0 (0.00%) | 0.49 |
| Table showing the differences in the Health workers involved in the composition of the MDT between north Italy and Central-South Italy ALS Centers.  Abbreviations: ALS, Amyotrophic Lateral Sclerosis; MV, Mechanical Ventilation; MDT, Multidisciplinary Team. | | | |
