# Supplementary material for: Withdrawal of mechanical ventilation in amyotrophic lateral sclerosis patients: a multicenter Italian survey
Source: Neurol Sci. 2023 Jul 7;44(12):4349–57. doi: 10.1007/s10072-023-06905-7 (PMC10641048; doi:10.1007/s10072-023-06905-7)
Supplement: Supplementary file 2 — Supplementary file2 (DOCX 31 KB) [file 10072_2023_6905_MOESM2_ESM.docx]

| Supplementary Material 2. Extended version of questions and answers of the survey. | | | |
| --- | --- | --- | --- |
|  | **Total answers (n= 38)** | | |
|  | **YES** | | **NO** |
| 1. Have you ever received a request for MV   withdrawal before or after Law 219/2017? | 22 (57.9%) | | 16 (42.1%) |
|  | **Before Law: 12**  **After Law: 10** | |  |
| 1-a. Was the MV withdrawal completed? | 16 (72.7%) | | 6 (27.3%) |
|  | **Before Law: 7**  **After Law: 9** | |  |
| 1-b. Was the request managed by a MDT? | 16 (72.7%) | | 6 (27.3%) |
| 1-c. Which was the setting of the MV  withdrawal? | Home, 9 (41.3%); Hospital, 7 (30.4%); Hospice, 6 (28.2%) | | |
| 1-d. What was the duration of the whole  procedure? | Mean: 3.6 ± 2.4 months; Interval: 1 week-6 months | | |
| 1-e. Which were the decisional steps  applied to manage the request? | Discussion with patients and family to assess the autonomy and awareness of the request, reassessment of the request after a variable time, collection of the patient’s wishes, MV withdrawal planning, 22 (100%); seeking medical-legal and ethical support before initiating the procedure, 2 (9.0%) | | |
|  | **YES** | **NO** | |
| 1. Have you ever taken part in a MV   withdrawal (as organizer, performer, or observer) before or after Law 219/2017? | 25 (65.7%) | | 13 (34.3%) |
| 2-a. In this case, was ACP previously  discussed? | 23 (92.0%) | | 2 (8.0%) |
|  | **Before Law: 7**  **After Law: 16** | |  |
| 1. Have you ever refused or deemed inappropriate a MV withdrawal request? | 0 (0.0%) | | 1. (100%) |
| 1. In the case of a MV withdrawal request, is there a MDT at your Center? | 27 (71.0 %) | | 1. (29 %) |
| 4.a- In this case, who are the members of the  MDT, apart from the neurologist? | Psychologist, 19 (70.3%); PM physician, 12 (44.4%); Pulmonologist, 9 (33.3%); Anaesthesiologist, 8 (29.6%); General practitioner, 6 (22.2%); Nurse, 7 (25.9%); Psychiatrist, 3 (11.1%); Medical and Legal Adviser of the hospital and Bioethicist, 2 (7.4%) | | |
| Table showing questions and answers of the survey on MV withdrawals in ALS patients in Italy.  Abbreviations: ALS, Amyotrophic Lateral Sclerosis; MV, Mechanical Ventilation; MDT, Multidisciplinary Team; ACP, Advance Care Planning; PM: Palliative Medicine. | | | |
